# African Centers of Excellence in Bioinformatics and Data Intensive Science: Building Capacity for Enhancing Data Intensive Infectious Diseases Research in Africa

**DOI:** 10.37191/mapsci-jidm-1(2)-006

**Published:** 2023

**Authors:** Maria Y Giovanni, Christopher Whalen, Darrell E Hurt, Latrice Ware-Allen, Karlynn Noble, Meghan McCarthy, Mariam Quinones, Phillip Cruz, Daudi Jjingo, Mamadou Wele, Doumbia Seydou, Michael Tartakovsky

**Affiliations:** 1 Office of Data Science and Emerging Technologies and Office of Cyber Infrastructure and Computational Biology, National Institute of Allergy and Infectious Diseases, National Institutes of Health, Bethesda, Maryland, USA; 2 Office of Cyber Infrastructure and Computational Biology, National Institute of Allergy and Infectious Diseases, National Institutes of Health, Bethesda, Maryland, USA; 3 Department of Computer Science, College of Computing and Information Sciences, and The African Center of Excellence in Bioinformatics and Data-Intensive Science, Infectious Disease Institute, Makerere University, Kampala, Uganda; 4 Institute of Applied Sciences, University of Sciences, Techniques and Technologies of Bamako, and The African Center of Excellence in Bioinformatics and Data-Intensive Science, Bamako; 5 Department of Public Health, Faculty of Medicine and Odontostomatology, University of Sciences, Techniques, and Technologies of Bamako, Bamako

**Keywords:** Training, Capacity building, Bioinformatics, Infectious diseases, Global health

## Abstract

Africa faces both a disproportionate burden of infectious diseases coupled with unmet needs in bioinformatics and data science capabilities which impacts the ability of African biomedical researchers to vigorously pursue research and partner with institutions in other countries.

The African Centers of Excellence in Bioinformatics and Data Intensive Science are collaborating with African academic institutions, industry partners, the Foundation for the National Institutes of Health (FNIH) and the National Institute of Allergy and Infectious Diseases (NIAID) at the National Institutes of Health (NIH) in a public-private partnership to address these challenges through enhancing computational infrastructure, fostering the development of advanced bioinformatics and data science skills among local researchers and students and providing innovative emerging technologies for infectious diseases research.

## Introduction

The emergence of innovative, genomic, and related-omics technologies has revolutionized how basic and clinical researchers are studying infectious diseases and vast amounts of diverse, large, and complex biomedical data are being generated at an unprecedented rate. Bioinformatics and data science-including powerful analytical tools-are providing tremendous opportunities for extracting knowledge from these data sets and accelerating research and discoveries in infectious diseases. Thus, increasing our understanding of pathogens and the diseases they cause impacts the development of new and improved therapeutic interventions and diagnostics. These incredible opportunities are aligned with challenges in storage, management, sharing, finding, visualizing, accessing, integrating, and analyzing data, and highlights the critical need for highly trained bioinformaticians, biostatisticians, and biomedical data scientists.

Despite significant advances and recognition that bioinformatics and data-intensive science are essential research tools and critical skills for biomedical researchers in their efforts to pursue basic and clinical research in infectious diseases, Africa faces both a disproportionate burden of infectious diseases and unmet needs in the bioinformatics and data science capabilities [1,2]. Challenges include limited access to reliable high-speed internet and a shortage of highly qualified bioinformaticians, statisticians, and data scientists. This impacts the use of available data resources because many data repositories and advanced computational tools and platforms are web-based and require strong and continuous internet connections, which are not always readily available or affordable in low resourced environments. Facility in using cyberinfrastructure and data analytic tools is a pre-requisite for innovative scientific inquiry and perhaps, more importantly, in supporting projects that rely on those computational platforms and tools.

Some efforts to address these challenges in Africa are ongoing and have served to advance the understanding of the nature and etiology of infectious diseases and to inform efforts to develop and provide effective therapeutic interventions; and to improve treatment, healthcare, surveillance, and public health responses including COVID-19 pandemic in Africa [1]. Over the last decade, support and commitments from African governments and international leadership, industry partners, and non-profit organizations have begun to address unmet needs in cyberinfrastructure, reliable power, high-speed internet, and research funding and build a cadre of trained bioinformatics and data scientists [3]. There have been significant efforts in providing high performance computing infrastructures in Africa, and academic institutions are offering bioinformatics training and degree-granting programs, some with global partnerships and support. For example, large-scale capacity building and research programs supported by the NIH include the NIAID’s West Africa International Center of Excellence in Malaria Research (ICEMR) and International Centers for Excellence in Research (ICERs) in Mali and Uganda focus on conducting basic and clinical research in infectious diseases and providing computational data resources and state of the art technologies. The Presidential Emergency Plan for AIDS Relief (PEPFAR) is another example of the dramatic impact that infrastructure development and bilateral engagement can have to dramatically improve research and health outcomes in low to middle-income countries [4]. These programs helped to lay the groundwork for the NIH Human Heredity and Health in Africa consortium (H3Africa) and the H3ABioNet Bioinformatics Network which has enhanced bioinformatics capacity, training, and infrastructure across the continent for managing, sharing, and accessing data; has stimulated increased collaborative research; and has developed bioinformatics training courses and curriculum [5,6].

NIH career development and bioinformatics training programs supporting global health and data science include the Fogarty International Center’s Nurturing Genomics and Bioinformatics Research Capacity in Africa (BRecA), the Eastern Africa Network for Bioinformatics Training (EANBiT), West African Center of Excellence for Global Health Bioinformatics Research Training program, and African Postdoctoral Training Initiative (APTI) in partnership with the Bill and Melinda Gates Foundation and the African Academy of Sciences. Industry programs such as IBM Research-Africa have worked across African countries to increase capabilities and training in data science and to promote data-driven strategies for healthcare in preventing, treating, and diagnosing infectious diseases. Investments by the World Bank such as the African Higher Education Centers of Excellence Project are also addressing national, regional, and local global health challenges and enhancing and building innovative capacities in training and research. The Developing Excellence in Leadership and Genetics Training for Malaria Elimination program in sub-Sahara Africa (DELGEME), sponsored by the Wellcome Trust in partnership with the Department of International Development, the Alliance for Accelerating Excellence in Science in Africa (AESA), and many other African partners, supports the development of analytical skills in bioinformatics, biostatistics, and emerging technologies for malaria control and elimination in sub-Saharan Africa. In addition, the establishment of the African Society for Bioinformatics and Computational Biology (ASBCB) has enabled the development of communities of bioinformaticians and data scientists across the continent.

## The African centers of excellence in bioinformatics and data-intensive science (ACE)

NIAID has significant experience and investment in supporting basic and clinical global research in infectious diseases and in building and sustaining partnerships, especially in low and low middle-income countries. These are key to fulfilling NIAID’s mission and to improving the health of the global community. As part of this investment, NIAID has a long-standing commitment to supporting bioinformatics and data resources such as computational infrastructure, data platforms and repositories, hands-on-bioinformatics training, and computational tools development to provide the basic and clinical research community with access to state of the art and innovative research data resources. These resources stand ready to assist in the event of emerging diseases, global pandemics, and health emergencies. This community of scientists and trainees can work with tools at the highest level using these established data resources on which to learn and hone their skills.

The achievements of NIAID’s ICERs in Africa, as mentioned above, coupled with serious burden of infectious diseases and unmet bioinformatics infrastructure and training needs in Africa were the foundation for the establishment of the NIAID ACE consortium [7]. Although NIAID supported genomics and bioinformatics centers and other related programs provide considerable opportunities in hands-on-training and workshops in bioinformatics and emerging technologies across the African continent, participants had limited access to computing infrastructure, high-speed, reliable internet, data analysis tools, and continuous training in their home institutions in Africa. To address these fundamental barriers for data-intense infectious research in Africa, ACE was established as a consortium of research and training centers facilitated by NIAID, in collaboration with African research and academic institutions, African governments, private sector companies, and the Foundation for the NIH (FNIH) through a public-private partnership. The goal of ACE is to improve access in Africa to computational capabilities and high performing computing infrastructure to enhance bioinformatics and data science training and mentorship. Thus, by providing and enhancing critical data resources and training in Africa, the goal is to empower biomedical researchers and students with access to state-of-the art computing capacity, data resources, and skills for data-intense research and data management and analysis. This, in turn, will help accelerate biomedical research and drive new discoveries that could impact the treatment, prevention, and diagnosis of diseases in Africa and across the globe. In addition, ACE provides an environment for local and regional African scientists to collaborate with researchers across the continent as well as the broader global biomedical research community.

The first ACE Center was established in Mali in 2015 as a public-private partnership between the University of Sciences, Techniques and Technologies of Bamako (USTTB)-who was launching a master’s graduate degree program-and with industry partners Intel Corporation, Hewlett Packard (through the Intel partnership), BioTeam, and EMC Corporation and the Foundation for the National Institutes of Health (FNIH). In 2019, a second ACE Center was opened in Uganda with Makerere University and the Infectious Diseases Institute (IDI), the Research and Educational Network of Uganda (RENU), the Texas Advanced Compute Center (TACC) and ENDUVO. Just as ACE-Mali serves as a regional bioinformatics resource center for research and academic institutions in West Africa, ACE-Uganda serves as a regional resource center for East Africa. ACE Centers offer high performance computing infrastructure for compute and storage capabilities; master’s and Ph.D. degree-granting programs in bioinformatics in collaboration associated with academic partners; telelearning classrooms with over 20 workstations; and superior audio, visual, and networking environments for enhancing virtual, collaborative global research with ACE researchers and students. ACE-Uganda piloted a visualization laboratory featuring virtual reality-based technology with six virtual reality (VR) workstations for research and training. A similar VR laboratory was set up at ACE-Mali in 2020.

## Partnerships and collaborative activities

The ACE Centers are built on a unique framework anchored in public-private partnerships that drive the support of computing infrastructure and bioinformatics training programs, both critical components for enhancing bioinformatics capabilities in Africa. This partnership allowed centers to be established, anchored by academic institutions with access to research space and environments, research and teaching faculty, degree-granting programs, and students interested in careers in bioinformatics. Industry partners provided in-kind contributions and donations for services, consulting, hardware, software configuration, internet connectivity, and software licenses. All the partners contribute to the Centers to support the objectives of ACE and to enhance research through education and infrastructure. For the universities in Mali and Uganda, their contributions included space in their institution’s buildings, renovated classrooms, and the faculty and staff to support the activities. U.S. partners contributed infrastructure, support services, and licenses for software used in the ACE Centers.

The ACE consortium is governed by the ACE Global Council, the Scientific Advisory Council, and the NIAID ACE Global Operations Team. The Global Council’s members are outstanding and well-respected scientists with diverse expertise in bioinformatics and data science, infectious diseases, and biotechnology and include non-voting members from the NIAID and ACE centers. Additionally, individual ACE Centers have local management teams to oversee the day-to-day centers’ operations. These are typically constituted by representatives from local collaborating institutions. As a partner, in addition to providing computational infrastructure, NIAID contributes scientific and technical staff to serve as members of the Scientific Advisory Council and Global Operations Team to assist the ACE Directors and faculty on Centers operations, teach courses and hands-on-workshops with faculty from African academic institutions, and provide scientific and technical support.

The ACE centers provide a research and training environment with state-of-the-art computational and training facilities that foster networking and collaborations to strengthen research activities. Networking and collaborative activities include hosting workshops and symposia. For example, ACE-Mali and ACE-Uganda have co-hosted workshops with MRC-University of Glasgow on viral bioinformatics. ACE-Mali held the 1st Congress of African Associations for Research and Control of Antimicrobial Resistance on-site with attendees from across the continent and hold an annual Global Health in Bioinformatic Symposium with a training bioinformatics workshop on advanced topics in data science. ACE-Uganda hosted data science workshops in collaboration with the African Center of Excellence in Materials, Products, and Nanotechnology at Makerere University and most recently provided a Virtual Reality demonstration at ACE-Uganda for participants of a workshop organized by Health Education England on Immersive Technology in Emergency Medicine. The ACE consortium supports rapid and timely sharing and access to diverse data sets generated by ACE Centers collaborative research projects and activities that promote the sharing of data in open access data repositories, where data are easy to find, accessible, and can be used and reused. ACE is committed to adhering to the F.A.I.R. principles for data sharing and access (Findable, Accessible, Interoperable, and Reusable) and policies and guidelines of the NIAID, the NIH, and other funding agencies, as appropriate [8,9]. These principles are necessary for accelerating research and discoveries and reducing the threat of infectious diseases globally.

## Bioinformatics and data science training

The ACE Centers offer bioinformatics and data science training programs and activities to enhance, foster, and sustain a diverse workforce of well-trained basic and clinical scientists with bioinformatics and computational expertise who can apply these skills to address global health issues in Africa. Sustaining a diverse workforce of well-trained scientists with bioinformatics and computational expertise and experience with advanced analytics such as artificial intelligence/machine learning will be dependent on increasing a wide range of training opportunities and degree-granting programs, and strong and committed mentors, coupled with career opportunities in Africa.

A multi-faceted training program supports master’s and Ph.D. degree-granting programs with academic partners USTTB, IDI, and Makerere University. Support includes curriculum and short and long course development, hands-on-train-the-trainer workshops, seminars, and lectures; all facilitated by ACE Centers’ state-of-the-art telelearning environments for in-person and remote and digital learning with dedicated access to high performance computing infrastructure and internet. A NIAID team of computational biologists, structural biologists, genomic scientists and others in collaboration with ACE faculty members and staff, develops and teaches courses in bioinformatics and emerging technologies as well as organizes workshops and seminars. Since 2017, almost 100 students from across the African continent have enrolled in ACE associated master’s and Ph.D. programs, and in ACE Mali 27 have completed advanced graduate degrees. Over two thirds of these MS graduates are either pursuing a PhD or have started working as Research Assistants in Malian laboratories and pursuing careers in data science or seeking advanced degrees. ACE-Uganda is currently supporting 58 Masters’ students and 9 Ph.D. students. It is anticipated that first cohort of students will be graduating in 2023 and some have already secured data science related positions. Most recently, ACE-Uganda has begun supporting postdoctoral fellows. The ACE consortium has developed and supported more than 200 on-site or remote bioinformatics lectures and hands-on-training on technical topics such as “Introduction to Linux” to introductory courses in bioinformatics, genomics, systems biology, structural biology, metabolomics, and proteomics. The students in both ACE Centers have been working on research topics that will have a direct impact on health, specifically on infectious diseases well known to their communities such as Malaria, Leishmaniasis, Retrovirus infection, and Tuberculosis. In addition, students are developing data analysis and bioinformatic workflows for broader use which leverages the knowledge acquired through ACE and compute infrastructure [10–13].

ACE-Uganda is providing the computational infrastructure and data resources to students, faculty, and researchers in support of the new bioinformatics training program, co-hosted by the Department of Immunology and Molecular Biology, College of Health Sciences and Department of Computer Science at the College of Computing and Information Science at Makerere University. The “Nurturing Genomics and Bioinformatics Research Capacity in Africa” (BRecA) program leverages highly skilled local and international faculty to increase the number of bioinformaticians in Africa and builds on the programs of the H3Africa-supported consortia (CAfGEN, TrypanoGEN, IBH3AU, and H3BioNET). The first cohort of the BReCA program enrolled 50 students and second cohort 24 additional students. In addition to access to the ACE infrastructure, the faculty of the BRecA program works closely with the ACE Global Operations Team to provide bioinformatics training for faculty through train-the-trainer workshops and developing and participating in remote hands-on courses and lectures for students. For example, ACE-Uganda is providing computational infrastructure and services to BRecA for data analysis to develop a software tool that provides HIV risk scores through a telehealth platform as part of funding from the Ugandan government.

ACE-Uganda hosted bioinformatics and data science training workshops, one with Makerere University and one with CUNY for postgraduate students and provided bioinformatics training on advanced computational tools related to areas such as structural biology and genomic data analysis. Currently, it is also supporting the development of the national sickle cell disease registry in collaboration with the Department of Pediatrics at Makerere University’s College of Health Sciences. ACE-Uganda has provided high performance computational infrastructure and access to develop and test a bioinformatics training/mentorship model that uses local computational infrastructure. It aims at supporting the faculty from Makerere University and enrich the curriculum with additional hands-on-skills training and mentorship and has successfully served to mentor students [14]. Since its inception, ACE-Mali has collaborated with and provided technical expertise and computational infrastructure to the Developing Excellence in Leadership and Genetics Training for Malaria Elimination program in sub-Sahara Africa (DELGEME). This training program is sponsored by the Well come Trust in partnership with the Department of International Development, the Alliance for Accelerating Excellence in Science in Africa (AESA), and many other African partners. DELGEME supports the development of analytical skills in bioinformatics, biostatistics, and emerging technologies for malaria control and elimination in sub-Saharan Africa. ACE Mali has a collaborative program with Tulane University focused on Master student training in Tulane University and ACE faculty training in curriculum development. ACE-Mali also hosted the NIH Fogarty International Center’s first symposium of the West African Center of Excellence for Global Health Bioinformatics Research Training in ACE’s telelearning facility, as well as a 10-day hands-on bioinformatics workshop on next generation sequencing and geographic information systems in collaboration with USTTB and Tulane University. The second symposium and bioinformatics workshop were held virtually in June 2021 with participants and lecturers from USA, France, India, Mali, and other African countries.

As an example of ACE Centers providing computational infrastructure and analysis to enhance research projects at partner academic institutions, one of ACE-Mali’s research collaborators used the data resources available at the ACE Center to support their clinical and genetic studies of hereditary neurological disorders (HND) in Mali. Facing several challenges in computational infrastructure as data storage and reliable internet and availability of bioinformaticians for data analysis, ACE-Mali provided infrastructure and technical bioinformatics support for data analysis.

## Computational infrastructure

Although there has been significant growth in Africa for enhancing computational infrastructure to accelerate biomedical research, significant gaps still exist that limit the ability of researchers and students to manage, use and re-use, access, and analyze data. The continuing lack of strong and reliable high-speed internet impacts the use of cloud-based data resources such as data repositories and advanced computational tools and platforms for data management and analysis. Technical support and maintenance are critical components in building a sustainable model for bioinformatics research and training centers in low-resource settings. The NIAID Global Operations team has been able to manage the infrastructure and classroom learning environments remotely using tools such as Git, Python, Salt and OpenStack to provide configuration management, versioning, and maintenance. The goal is to build sustainable support systems in Africa for training and research–working towards the goal of infrastructure-as-code (IAC) that can allow configuration management and system administration in cloud platforms. The infrastructure leverages cloud operating systems to provision not just to the ACE Centers, but also cloud platforms such as Amazon Web Services, Google, or Microsoft Azure Stack. With this new infrastructure, ACE collaborators, students, and scientists have the data resources to develop and run scientific computing tools for data analytics needed to keep local research current and competitive.

Over the last six years, the ACE Centers have become regional bioinformatics centers to support biomedical researchers and students. Using in-kind donations from public-private partnerships, the Centers are equipped and managed with state-of-the-art computational infrastructure that includes strong compute cores, high speed internet access, and a telelearning center with more than 20 stations. ACE-Uganda’s cutting edge biomedically-focused virtual reality technology is available for research and training with six Virtual Reality (VR) stations and used to enhance learning and training for students and faculty. During the COVID-19 pandemic, ACE-Uganda took advantage of the VR stations and equipment and developed a training course in Infection, Prevention, and Control (IPC) which included using personal protection equipment (PPE) for frontline healthcare workers [15]. The local exposure and training of these technologies provides opportunities for researchers to explore new ways to look at data, training, and remote collaborations in the VR facility.

## Conclusions and looking ahead

The ACE consortium serves as a model using a public-private partnership with industry, academic institutions, African governments and other governments as U.S. and non-profit organizations work together to address unmet needs in bioinformatics capacity in Africa and is uniquely poised to impact the training the next African generation of scientists in bioinformatics that are highly skilled to address key research questions in infectious diseases. The partnership includes the use of research facilities and degree-granting programs in academic institutions in Africa, in-kind donations from industry for consulting and computational infrastructure, the Foundation for the National Institutes of Health (FNIH) to facilitate the public-private partnership model, and NIAID’s contribution of scientific and technical staff to assist the ACE Directors and faculty on day-to-day centers operations, teaching courses with faculty from African academic institutions, and provide considerable technical support.

Despite the accomplishments of the ACE consortium and other related programs in Africa in reducing barriers in bioinformatics and data science capabilities by improving access to high performance computing and reliable internet, enhancing bioinformatics and data science training and mentorship, increasing the number of trained bioinformaticians in Africa, and advancing biomedical research, much remains to be done. Academic and research centers with strong bioinformatics capacity and training programs exists, yet data research resources and training are not consistently available across the African continent. The sustainability of the ACE Consortium is of highest priority to ensure continuous operation and provide innovative and state-of-the-art facilities and computational capabilities. Key to sustainability will be to enhance local capabilities and funding at ACE Centers for infrastructure, training, and faculty research projects and for the ACE Centers to develop local and international partnerships and seek funding opportunities from local governments, associated academic institutions, local industry, funding agencies, and nonprofit organization to support ACE consortium.

The availability and accessibility of data research resources and bioinformatics training for African biomedical researchers and students as they tackle data-intense research projects is essential to leveraging data-driven strategies and transforming data into knowledge, thus impacting the development of new and improved therapeutic interventions, prevention strategies, and diagnostics to reduce the threat of infectious diseases and improve health care in Africa. These efforts need to be expanded across the continent and the key is sustainability through continuous commitment and funding to maintain state-of-the-art computational infrastructure and other data research resources and bioinformatics training programs.
